# Combined Superbase Ionic Liquid Approach to Separate CO_2_ from Flue Gas

**DOI:** 10.1021/acssuschemeng.2c01848

**Published:** 2022-07-13

**Authors:** Adam J. Greer, S. F. Rebecca Taylor, Helen Daly, Johan Jacquemin, Christopher Hardacre

**Affiliations:** † Department of Chemical Engineering, The University of Manchester, The Mill, Sackville Street, Manchester M13 9PL, U.K.; ‡ Université de Tours, Laboratoire PCM2E, Parc de Grandmont, 37200 Tours, France; § Materials Science and Nano-Engineering, Mohammed VI Polytechnic University, Lot 660-Hay Moulay Rachid, Ben Guerir 43150, Morocco

**Keywords:** ionic liquids, CO_2_ capture, SO_2_ capture, flue gas, scrubber

## Abstract

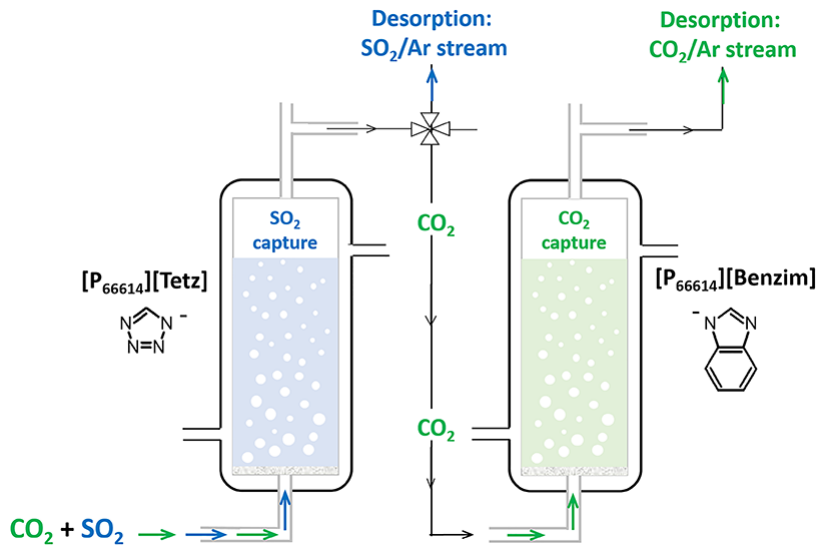

Superbase ionic liquids (ILs) with a trihexyltetradecylphosphonium cation and a benzimidazolide ([P_66614_][Benzim]) or tetrazolide ([P_66614_][Tetz]) anion were investigated in a dual-IL system allowing the selective capture and separation of CO_2_ and SO_2_, respectively, under realistic gas concentrations. The results show that [P_66614_][Tetz] is capable of efficiently capturing SO_2_ in preference to CO_2_ and thus, in a stepwise separation process, protects [P_66614_][Benzim] from the negative effects of the highly acidic contaminant. This results in [P_66614_][Benzim] maintaining >53% of its original CO_2_ uptake capacity after 30 absorption/desorption cycles in comparison to the 89% decrease observed after 11 cycles when [P_66614_][Tetz] was not present. Characterization of the ILs post exposure revealed that small amounts of SO_2_ were irreversibly absorbed to the [Benzim]^−^ anion responsible for the decrease in CO_2_ capacity. While optimization of this dual-IL system is required, this feasibility study demonstrates that [P_66614_][Tetz] is a suitable sorbent for reversibly capturing SO_2_ and significantly extending the lifetime of [P_66614_][Benzim] for CO_2_ uptake.

## Abstract 2

Feasibility study for the use of ILs in a stepwise separation process showing [P_66614_][Tetz] to increase the lifetime of [P_66614_][Benzim] as a CO_2_ capture sorbent.

## Introduction

Gas capture sorbents such as alkanolamines, metal–organic frameworks, porous liquids, and ionic liquids (ILs) are being increasingly studied in the literature for CO_2_ uptake to combat rising greenhouse gas emissions.^1−6^ While CO_2_ emissions are subject to legislation in the UK, in line with achieving net zero by 2050, other components of waste gas streams such as SO_2_ and nitrogen oxides (NO_*x*_) also require removal due to their contribution to acid rain and smog formation.^7^

For CO_2_ removal from flue gas streams, aqueous alkanolamine scrubbers are the benchmark carbon capture technology commercially available. The production and regeneration of these sorbents are energy intensive, and the loss of the amine sorbent during operation, due to degradation and evaporation, brings additional environmental considerations along with the production of highly corrosive waste solutions.^8^ In terms of CO_2_ capture, the addition of contaminants such as SO_2_ has been demonstrated to degrade current capture sorbents like alkanolamines; however, consideration of the effect of such impurities has not been widely considered in the literature when investigating the viability of new CO_2_ capture sorbents.^9−11^

Currently, approximately 85% of power plant desulfurization occurs via a wet limestone–gypsum process which has a high desulfurization rate and a low cost of operation.^8,12,13^ This process requires waste water treatment, and while gypsum (formed as a by-product) has a number of applications such as in wallboard manufacturing and the cement industry, globally, a large proportion goes to landfill.^14,15^ The efficiency of such desulfurization processes (and other purification stages) before CCS (Carbon Capture and Storage) significantly impacts the sorbent lifetime and the overall process efficiency and cost.

ILs have received substantial interest for gas capture due to a tunable structure through the pairing of different cations and anions and favorable physical properties (high thermal stability, low melting point, and low vapor pressure).^16^ In addition, reported calculations show reductions in energy consumption (lower temperature/pressure sorbent regeneration conditions) and overall costs for IL-based gas separation processes, compared to commercial amine-based CO_2_ absorbents, which are additional drivers for their use.^17,18^ The ability of an IL to interact either physically or chemically with different gases allows the process to be tailored to optimize the gas uptake capacities through a knowledge of the absorption enthalpies and physiochemical properties of the ILs.^19−21^ Therefore, the available data can now be used to identify a wide range of structure–activity relationships for selective gas capture by ILs.^17,22−24^

In the majority of cases, the anion has been shown to have the largest effect on gas uptake capacities, where there is now an increasing trend in investigating the consequence of altering the anion basicity.^20,25,26^ In particular, superbase ILs (SBILs) with aprotic heterocyclic anions have been shown to reversibly capture large amounts of CO_2_ (>1 *n*CO_2_/*n*IL) with minimal increases in viscosity,^27,28^ an important consideration in the industrial application of ILs. For SBILs, where it has been demonstrated that the basicity of the anion can affect the absorption enthalpies, the presence of acidic gases such as SO_2_ in the feed can significantly affect the recyclability of the sorbent.^29−32^

The effect of the anion on the capture of CO_2_ and SO_2_ is displayed in Table 1 for two SBILs: trihexyltetradecylphosphonium benzimidazolide, [P_66614_][Benzim], and trihexyltetradecylphosphonium tetrazolide, [P_66614_][Tetz]. [P_66614_][Benzim] has been used in a series of studies investigating the effect of flue gas contaminants on CO_2_ uptake and was also shown to capture significantly more CO_2_ per mole of IL in comparison to [P_66614_][Tetz] (1.20 *vs* 0.08 *n*CO_2_/*n*IL) due to a higher absorption enthalpy (−52.1 *vs* −19.1 kJ·mol^–1^).^20,27,31−34^ Importantly, when SO_2_ capture is considered, both ILs can absorb large amounts. However, [P_66614_][Benzim] does so irreversibly, which has been shown to have a negative effect on its ability to simultaneously absorb CO_2_.^32,33^ In contrast, a small residue following desorption (0.06 *n*SO_2_/*n*IL) was reported for [P_66614_][Tetz] after exposure to 0.2 vol % SO_2_ which may be due to a weaker binding affinity when compared to [P_66614_][Benzim] (−89.4 *vs* −123.9 kJ·mol^–1^).^31−33^ However, a study of the recyclability of [P_4442_][Tetz] showed that the working absorption capacity remained constant.^31^

**Table 1:**
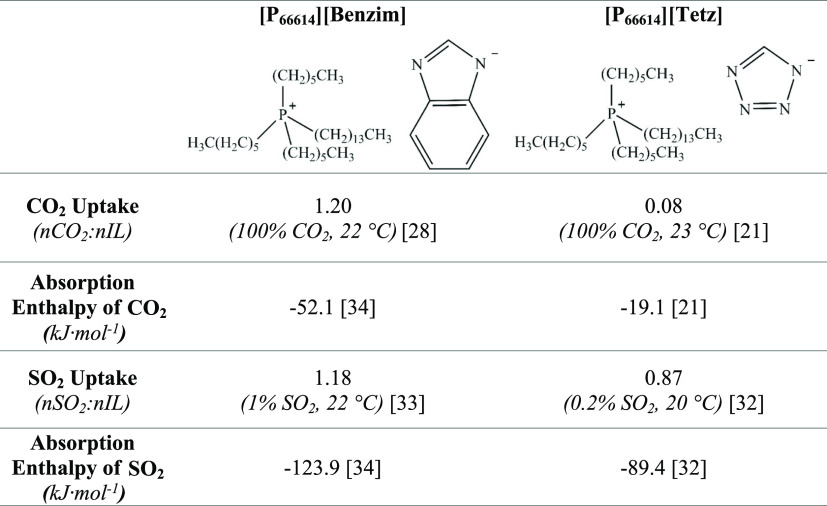
CO_2_ Capacity, SO_2_ Capacity, and Absorption Enthalpy of the Two SBILs Investigated in This Work; Trihexyltetradecylphosphonium Benzimidazolide, [P_66614_][Benzim], and Trihexyltetradecylphosphonium Tetrazolide [P_66614_][Tetz]

The differing absorption enthalpies of these SBILs toward CO_2_ and SO_2_ could allow the possibility of using the two SBILs in series for stepwise gas separation, with [P_66614_][Tetz] used to selectively absorb SO_2_. In this case, [P_66614_][Tetz] acts as a scrubber and extends the lifetime of [P_66614_][Benzim] for the reversible capture of CO_2_. Previous studies on the gas absorption of SO_2_ by [P_66614_][Benzim] showed that in the presence of realistic flue gas amounts of SO_2_ (0.2 vol %), an 89% decrease in CO_2_ capacity was observed over 11 absorption/desorption cycles.^32^

Herein, the feasibility of using [P_66614_][Tetz] with [P_66614_][Benzim] in a dual-IL system has been investigated to assess the effect on the lifetime and recyclability of the [P_66614_][Benzim] sorbent for CO_2_ uptake. This study has utilized the previously developed mass spectrometry-based gas absorption rig,^32–34^ with the ILs after exposure characterized using NMR and elemental analyses to determine their recyclability. This paper is a feasibility study for the use of ILs in a stepwise separation process and shows [P_66614_][Tetz] to increase the lifetime of [P_66614_][Benzim] as a CO_2_ capture sorbent.

## Experimental Section

### Materials

Trihexyltetradecylphosphonium chloride (97.7 wt %, CAS: 258864-54-9) was obtained from IoLiTec; benzimidazole (98 wt %, CAS: 51-17-2) and tetrazole (CAS: 288-94-8) were purchased from Sigma-Aldrich. [P_66614_][Superbase] was prepared using a previously reported two-step synthesis method.^27^ The structure and purity of the ILs, after synthesis and post-exposure, were analyzed using ^1^H NMR and ^13^C NMR with a Bruker AVANCE II 500 MHz Ultra shield Plus spectrometer and carried out as neat ILs in the presence of a glass capillary insert containing DMSO-*d*_6_, purchased from Cambridge Isotope Laboratories Inc. (CAS: 2206-27-1). The halide content was determined to be <5 ppm using a silver nitrate test, and water content was measured to be <0.1 wt % using a Metrohm 787 KF Titrino Karl Fischer machine. The following gases were obtained from BOC: argon (99.998%, CAS: 7440-37-1), carbon dioxide (99.99%, CAS: 124-38-9), and sulfur dioxide (1% in argon, CAS: 7446-09-5).

The IL samples post exposure to the gases were stored in an argon-filled glovebox before analysis. Elemental analysis was carried out using a Thermo Scientific Flash 2000 elemental analyzer.

### Methods

**Figure 1:**
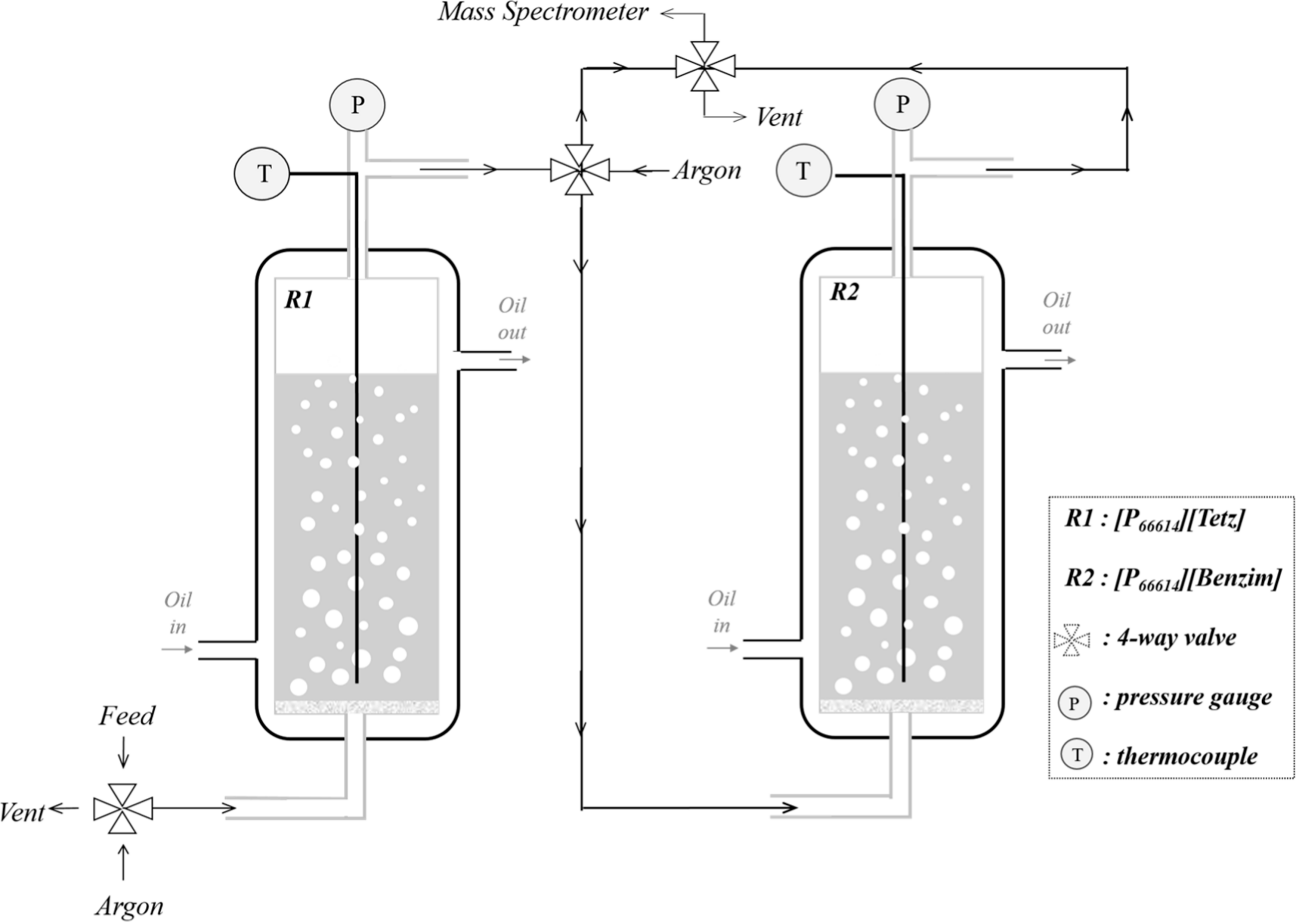
Schematic depicting the gas absorption rig used in this work.

In previous work, a novel gas absorption technique utilizing mass spectrometry was developed to allow the study of mixed component gas feeds that resemble realistic flue gas conditions.^32−34^ This experimental setup was modified, herein, to allow the inclusion of two reactors connected in series, and this is shown in Figure 1. ∼2 g (±0.1 mg) of [P_66614_][Benzim] and [P_66614_][Tetz] were weighed out separately in an argon-filled glovebox and transferred into individual temperature-controlled glass reactors. A series of absorption and desorption cycles were then carried out consisting of a 2 h absorption period at 22 °C (±0.05 °C) and 1 atm (±0.05 atm) under feed conditions of 14% CO_2_ in Ar or 14% CO_2_ + 0.2% SO_2_ in Ar, followed by a 2 h desorption step at 90 °C under Ar.

To prevent the exposure of [P_66614_][Benzim] to SO_2_ during the desorption step, a four-way valve was used to direct the outlet from the reactor containing [P_66614_][Tetz] to the vent, while the outlet of [P_66614_][Benzim] was directed to the mass spectrometer. Initially, a baseline for the absorption of CO_2_ by the two ILs was found by flowing a feed of 14% CO_2_ in Ar through both reactors consecutively. Six cycles were performed, yielding an average CO_2_ uptake of 0.77 *n*CO_2_/*n*IL, agreeing with previously published work. Three cycles with a feed of 14% CO_2_ in Ar were then carried out after every five consecutive 14% CO_2_ + 0.2% SO_2_ in Ar cycles to monitor the CO_2_ capacity of the ILs. The breakthrough curve from the mass spectra was used to calculate the CO_2_ uptake with an error of ±0.04 *n*CO_2_/*n*IL.

## Results and Discussion

**Figure 2:**
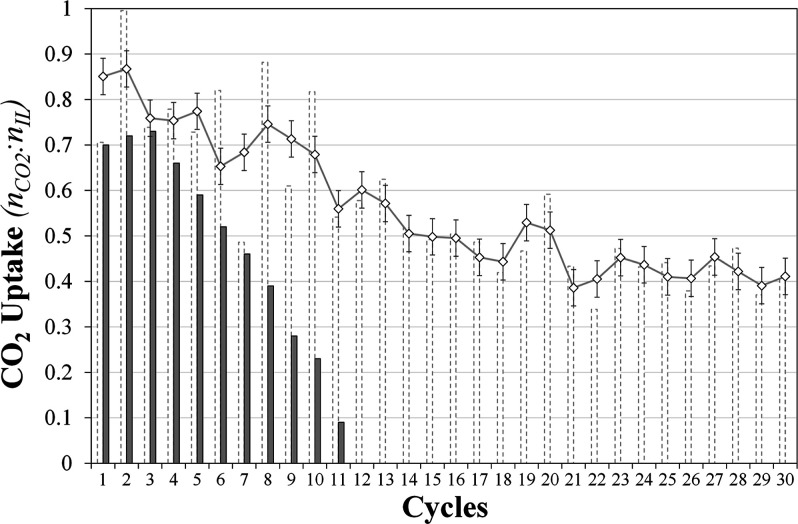
CO_2_ capacity of [P_66614_][Benzim] with (dashed bars) and without the presence of [P_66614_][Tetz] (filled bars) after 30 cycles of 2 h absorption at 22 °C under a feed of 14% CO_2_ and 0.2% SO_2_ in Ar and a 2 h desorption at 90 °C under Ar. The diamonds depict a moving average of subsequent CO_2_ uptake values, with an error of ±0.04 *n*CO_2_/*n*IL. Data for the CO_2_ capacity of [P_66614_][Benzim] without [P_66614_][Tetz] is available from ref (32).

The gas absorption rig developed was used to determine whether the use of [P_66614_][Tetz] as a SO_2_ scrubber would protect the CO_2_ recyclability of [P_66614_][Benzim] under a realistic gas feed (14% CO_2_ + 0.2% SO_2_ in Ar). It should be noted that the design of the scrubber was not optimized to maximize SO_2_ removal in this feasibility study. The results are shown in Figure 2 and are presented in comparison to earlier work without the presence of the [P_66614_][Tetz] scrubber, where it was shown that SO_2_ irreversibly absorbs to the [Benzim]^−^ anion resulting in the formation of an irreversibly absorbed sulfur species and deactivation of the IL to CO_2_ capture.^32^

Initial observations revealed that the CO_2_ capacity of [P_66614_][Benzim] dropped from 0.77 to 0.71 *n*CO_2_/*n*IL after one cycle under the CO_2_ + SO_2_ in Ar feed. As this is outside the error of the measurement (0.04 *n*CO_2_/*n*IL), the decrease could either be attributed to the influence of SO_2_ on [P_66614_][Benzim] and/or the loss of a small CO_2_ contribution from [P_66614_][Tetz] due to the stronger absorption enthalpy of SO_2_. This is because the initial CO_2_ only value of 0.77 *n*CO_2_/*n*IL includes contributions from both [P_66614_][Benzim], and [P_66614_][Tetz]. As the number of cycles increases, both ILs are exposed to increasing amounts of SO_2_, and it can be seen that the CO_2_ capacity of [P_66614_][Benzim] decreases further. For this 1:1 ratio of [P_66614_][Tetz] to [P_66614_][Benzim] (by mass), the decreasing CO_2_ capacity of [P_66614_][Benzim] suggests that the reactor containing [P_66614_][Tetz] was unable to fully remove SO_2_ from the mixed gas feed and allowed the exposure of a reduced amount of SO_2_ to interact with [P_66614_][Benzim].

After 11 absorption/desorption cycles, the CO_2_ capacity of [P_66614_][Benzim] had reduced to 0.54 *n*CO_2_/*n*IL, a 30% decrease. Conversely, when the gas feed was not scrubbed by [P_66614_][Tetz], an 89% decrease (to 0.09 *n*CO_2_/*n*IL) in the CO_2_ capacity of [P_66614_][Benzim] was observed, demonstrating the significant impact [P_66614_][Tetz] has on extending the lifetime of the sorbent.^32^ As such, a total of 30 cycles were carried out on the new system, after which it was found that [P_66614_][Benzim] still maintained >53% of its original capacity (0.41 *n*CO_2_/*n*IL). For comparison, the theoretical maximum CO_2_ uptake by the benchmark alkanolamine sorbent is 0.5 *n*CO_2_/*n*MEA.^35^ The CO_2_ uptake capacity demonstrated herein shows potential for dual-IL systems even after numerous regenerative treatments.

Additionally, it can be seen that after 21 cycles, the CO_2_ uptake began to stabilize, suggesting that an equilibrium point was reached. It is possible that at these extremely low concentrations of SO_2_ and relatively much higher concentrations of CO_2_, CO_2_ can compete for the absorption sites within the [P_66614_][Benzim] IL.

Previous work with NO_*x*_ and SO_2_ demonstrated that acidic gases can physically and chemically interact with both N-sites on the [Benzim]^−^ anion, suggesting that under these conditions SO_2_ could feasibly be strongly absorbed to one N-site, while CO_2_ is more weakly absorbed on the second N-site and competes with the lower SO_2_ concentration in the feed.^33,34^ It is important to note that similar studies have shown that in the presence of the [Tetz]^−^ anion, SO_2_ can interact on multiple N-sites. In this case, the absorption enthalpies decrease as subsequent absorption sites are filled.^29^

Further analysis of the results revealed fluctuations between cycles in the calculated CO_2_ capacities; however, the overall trend was still exposed. The results were plotted with a moving average (indicated by diamonds in Figure 2), and this showed that the variations decrease as the number of cycles increases. After every five CO_2_/SO_2_ absorption/desorption cycles, three CO_2_ only cycles were performed, and the average CO_2_ uptake is displayed in Figure S1. This shows further agreement with the experimental results, where a decrease in CO_2_ uptake occurs, followed by stabilization after 20 CO_2_/SO_2_ cycles (0.41 *n*CO_2_/*n*IL).

The theoretical absorption enthalpies for [P_66614_][Tetz] suggest that this IL will preferably absorb SO_2_ (−89.4 kJ·mol^–1^) over CO_2_ (−19.1 kJ·mol^–1^).^20,31^ To explore this, the mass spectrometry-derived breakthrough curves for SO_2_ are plotted in Figure S2. This shows that for at least the first 20 cycles, an extremely low (unmeasurable) amount of SO_2_ exits the reactors, indicating that the vast majority of the SO_2_ present in the feed is being captured by both ILs. Over the next 10 cycles, the breakthrough of SO_2_ was observed indicating accumulation of SO_2_ absorbed in the IL and that a saturation point had been reached for some of the absorption sites. Interestingly, this correlates with an equilibrium being reached in terms of CO_2_ uptake, suggesting that there is competition for the absorption sites.

At this stage, it is also important to note that during one absorption cycle, [P_66614_][Tetz] is exposed to a calculated 0.12 *n*SO_2_/*n*IL and therefore does not exceed the overall capacity of the IL (0.87 *n*SO_2_/*n*IL).^31^ With optimization of the scrubber design, for example, reducing the bubble size, higher levels of SO_2_ uptake would be achievable at lower amounts of IL, which would be favorable to the process costs. Subsequent characterization of [P_66614_][Tetz] (below) indicates that the absorption of SO_2_ is reversible and demonstrates that the detection of increased amounts of SO_2_ by mass spectrometry is not caused by incomplete regeneration of the sorbent but may be due to a loss of efficiency due to the ability of [P_66614_][Tetz] to absorb gases on multiple absorption sites with differing binding affinities.^31,36^

The influence of residual water, present in either the ILs or gas feed, was also considered as a potential mode of deactivation of the ILs as this can lead to the formation of further acidic gas species or protonation of the IL anion.^37^ However, no significant impact from water was observed in this work.

**Figure 3:**
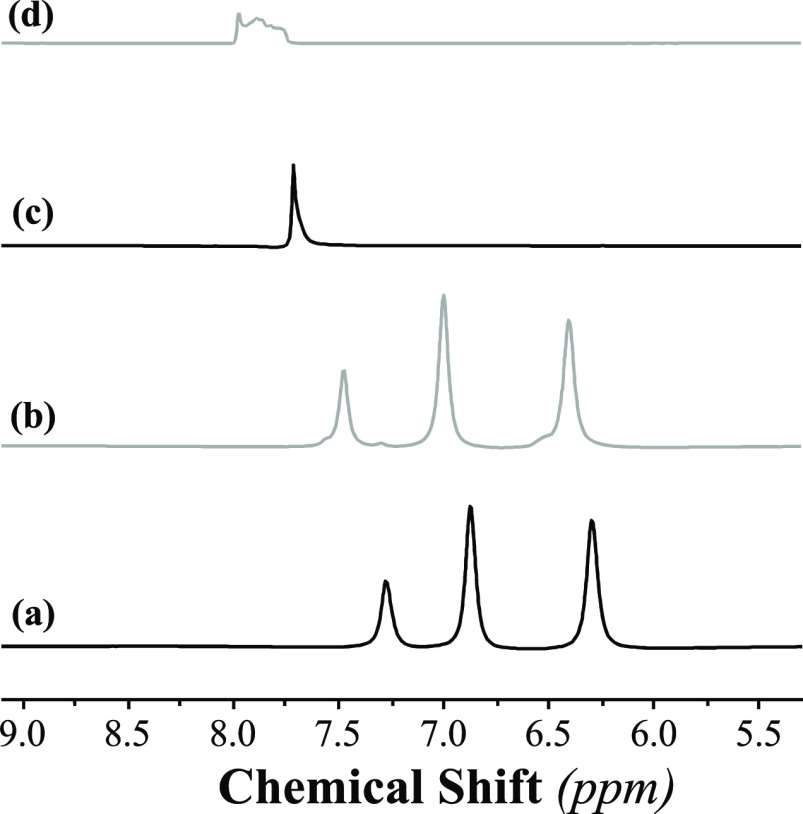
^1^H NMR spectra of [P_66614_][Benzim] before (a) and after (b) exposure and [P_66614_][Tetz] before (c) and after (d) exposure to the CO_2_ and SO_2_ absorption cycles.

The ILs were subsequently removed from the gas absorption rig after 30 cycles and characterized using NMR and elemental analyses. ^1^H NMR indicates a small downfield shift in the protons attached to the [Benzim]^−^ anion (Figures 3 and S3) from 6.30/6.88/7.28 to 6.41/7.00/7.48 ppm. This agrees with previous literature and indicates the irreversible absorption of a small amount of SO_2_, as expected from the gas absorption rig results.^30,32^ In the case of the [Tetz]^−^ anion, a downfield shift was observed from 7.72 to 7.97 ppm, as well as a broadening of the peak. These studies have noted that there is often a desorption residue (0.06 *n*SO_2_/*n*IL) for [P_66614_][Tetz], indicating the irreversible absorption of a small amount of SO_2_ and explaining the small chemical shift changes; however, a significantly high recyclability was still observed.^29,31^ The ^13^C NMR spectra are shown in Figure S3 where shifts were also found for the anionic peaks, agreeing with these findings.

Elemental analysis (Table S1) was performed on both ILs before and after exposure in the gas absorption rig, where [P_66614_][Benzim] was found to show a small increase in the sulfur content (to 1.64 wt %). This is in comparison to 4.56 wt % that was found in previous work after 11 cycles with no [P_66614_][Tetz] scrubber, demonstrating a 64% decrease in the sulfur content in [P_66614_][Benzim] after 30 cycles.^32^ In contrast, for [P_66614_][Tetz], no change was observed after exposure, in agreement with the indication that the SO_2_ is reversibly absorbed in this IL. It should be noted that a desorption residue has been observed by other authors and in the NMR results, herein, but was not observed in the elemental analysis results.^31^ It is possible that this is a result of the uncertainty from the elemental analyzer used in this work (*u* = 0.3 wt %) as the contamination is expected to be low from previous studies.^31^

The post-exposure characterization reveals that the use of [P_66614_][Tetz] as a reversible scrubber to remove SO_2_ from the feed and protect [P_66614_][Benzim] was not completely effective, with the results indicating the incorporation of a small amount of SO_2_ into the structure of [P_66614_][Benzim] and a subsequent decrease in the CO_2_ capacity. Optimization of the dual-IL system in terms of the ratio of [P_66614_][Tetz] to [P_66614_][Benzim] and the flow rate/residence time of the gases in the ILs could feasibly increase the SO_2_ uptake and, therefore, the CO_2_ uptake and lifetime of [P_66614_][Benzim], improving the overall efficiency of the process for longer-term operation. Furthermore, stepwise processes for the capture and separation of SO_2_ and CO_2_ have been reported to save ∼50% of the energy compared to simultaneous separation processes.^38^

In addition, such a dual-IL system, with stepwise separation of SO_2_ and CO_2_ from flue gas, could provide pure gas streams (albeit diluted in an inert gas) during desorption and regeneration of the IL. The pure SO_2_ gas streams could be utilized as feedstocks for processes such as conversion to elemental sulfur and/or used in sulfuric acid production,^39^ and the CO_2_ stream could be used in the production of chemicals and fuels such as urea, a major agricultural fertilizer, or methane (dry reforming or hydrogenation), as well as in the food and beverage industries.^40^ In situ utilization of gases absorbed (and activated) by ILs is also a continually developing research area.^41−45^ However, barriers to the deployment of IL systems for gas capture include their high viscosity (with consideration to viscosity changes upon gas absorption required) and the cost of IL production on an industrial scale. In the development of such task-specific ILs for gas separation processes, the toxicology and environmental impact of the chosen ILs require evaluation alongside the energy consumption and techno-economic calculations/modeling of the desired process.^4,46^

This technology has the potential to be applied to NO_*x*_ removal, as well as SO_2_. A number of ILs, including [P_66614_][Tetz], have been shown to display large “working” capacities for NO, presenting an opportunity for removing both contaminants simultaneously through multisite absorption capability.^36,47−51^ However, it is essential to study conditions that are representative of real-world environments to assess the impact of realistic amounts of flue gas contaminants on the ability of IL sorbents to reversibly capture CO_2_.^32−34^ Gas capture in thin films of ILs, such as [P_66614_][Benzim], is also being investigated as studies have shown that their behavior differs in comparison to bulk solution.^52^

Finally, it is important to consider the advantages and disadvantages that ILs present in comparison to other CO_2_ capture solvents for a commercial process, both in terms of performance and also economic feasibility, presenting an area for future exploration.^5,16^

## Conclusions

This work has demonstrated that the use of [P_66614_][Tetz] as a SO_2_ scrubber enables the extended lifetime of a CO_2_ capture sorbent, [P_66614_][Benzim], where after 30 absorption/desorption cycles, >53% of its original CO_2_ uptake capacity remains. Further design engineering offers the potential to limit the degradation of [P_66614_][Benzim] by increasing the efficiency of SO_2_ removal. Deactivation of [P_66614_][Benzim] to the absorption of CO_2_ was characterized using NMR and elemental analyses, showing the irreversible uptake of sulfur into the IL, blocking the active sites. However, analysis of [P_66614_][Tetz] showed that this IL was largely unaffected by SO_2_ absorption, offering the capability of reversibly absorbing SO_2_ under the studied conditions.

By tuning the basicity of the anion ([Tetz]^−^) to initially and selectively capture SO_2_, an anion with a higher affinity for gas capture ([Benzim]^−^) was able to be used to remove CO_2_ from a multicomponent gas feed. Anion effects have been well investigated for gas capture, but this is the first time, to the best of the authors’ knowledge, that a dual system has been proposed and investigated. However, it is also important to consider the effect of the cation on basicity by tuning the cation–anion interactions, and this offers an area for continued exploration.^53^

## Supporting Information Available

Averaged CO_2_ uptakes; mass spectrometry breakthrough curves for SO_2_; ^1^H NMR; ^13^C NMR; and elemental analysis of ILs before and after exposure (PDF)
