# Supplementary material for: Combined Superbase Ionic Liquid Approach to Separate CO2 from Flue Gas
Source: ACS Sustain Chem Eng. 2022 Jul 13;10(29):9453–9. doi: 10.1021/acssuschemeng.2c01848 (PMC9326967; doi:10.1021/acssuschemeng.2c01848)
Supplement: Supplementary file 1 — sc2c01848_si_001.pdf [file sc2c01848_si_001.pdf]

Combined Superbase Ionic Liquid Approach to Separate CO_2 from Flue Gas

Adam J. Greer[1,], S. F. Rebecca Taylor[1], Helen Daly[1], Johan Jacquemin[2,3,]*, Christopher Hardacre[1,]**

[1] Department of Chemical Engineering and Analytical Science, The University of Manchester, The Mill, Sackville Street, Manchester, M13 9PL, United Kingdom

[2] Université de Tours, Laboratoire PCM2E, Parc de Grandmont, 37200, Tours, France

[3] Materials Science and Nano-Engineering, Mohammed VI Polytechnic University, Lot 660-Hay Moulay Rachid, Ben Guerir, 43150, Morocco

AUTHOR INFORMATION

Co-Corresponding Authors:

* Christopher Hardacre, Tel: +44 (0) 161 306 2672, E-mail: c.hardacre@manchester.ac.uk

* Johan Jacquemin, Tel: +212 (0) 666 933 996, E-mail: johan.jacquemin@um6p.ma

* Adam Greer, Tel: +44 (0) 161 306 2227, E-mail: adam.greer@manchester.ac.uk

Number of pages: 8

Number of Figures: 4

Number of Tables: 1

Contents:

Average CO_2 uptake values - Figure S1 page S3

Mass spectrometry breakthrough curves for SO_2 - Figure S2 page S4

^{1}H NMR data - Figure S3 page S5

^{13}C NMR data - Figure S4 page S7

Elemental analysis of ILs before and after exposure - Table S1 page S8

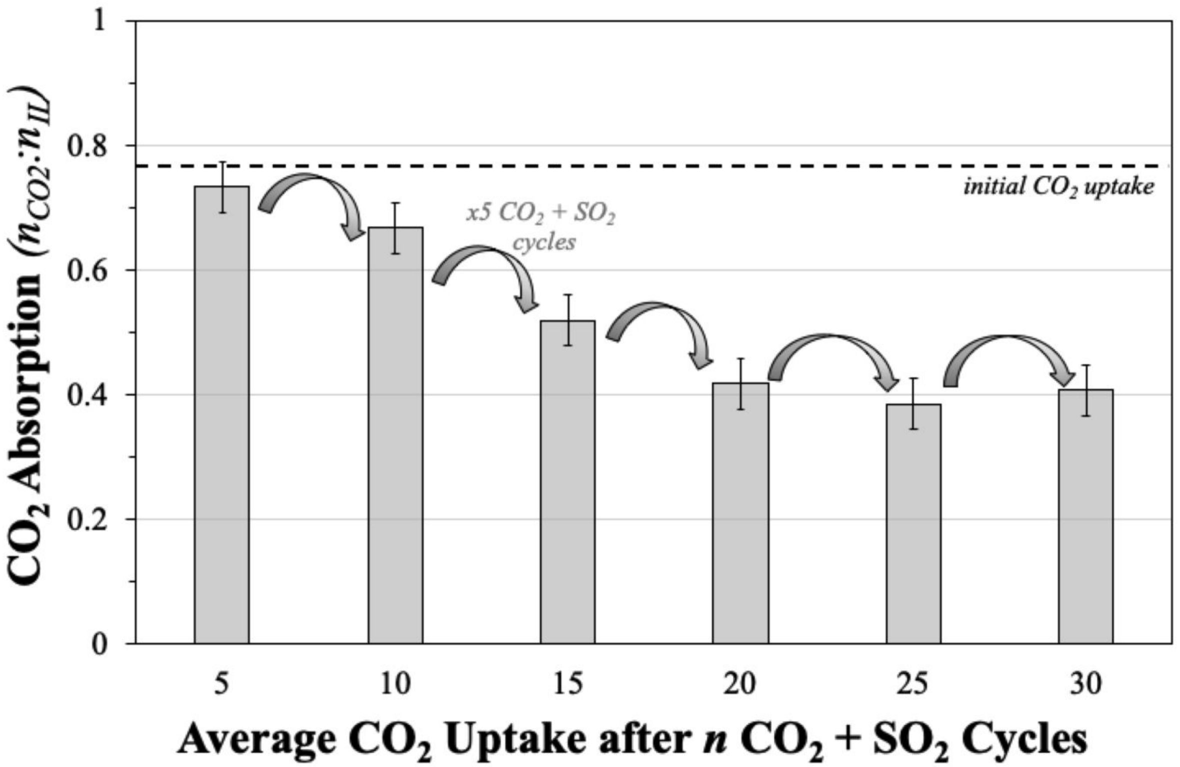

Figure S1. Depicts the average CO_2 uptake from three consecutive 14% CO_2 only cycles, carried out every five consecutive 14% CO_2 + 0.2% SO_2 in Ar cycles.

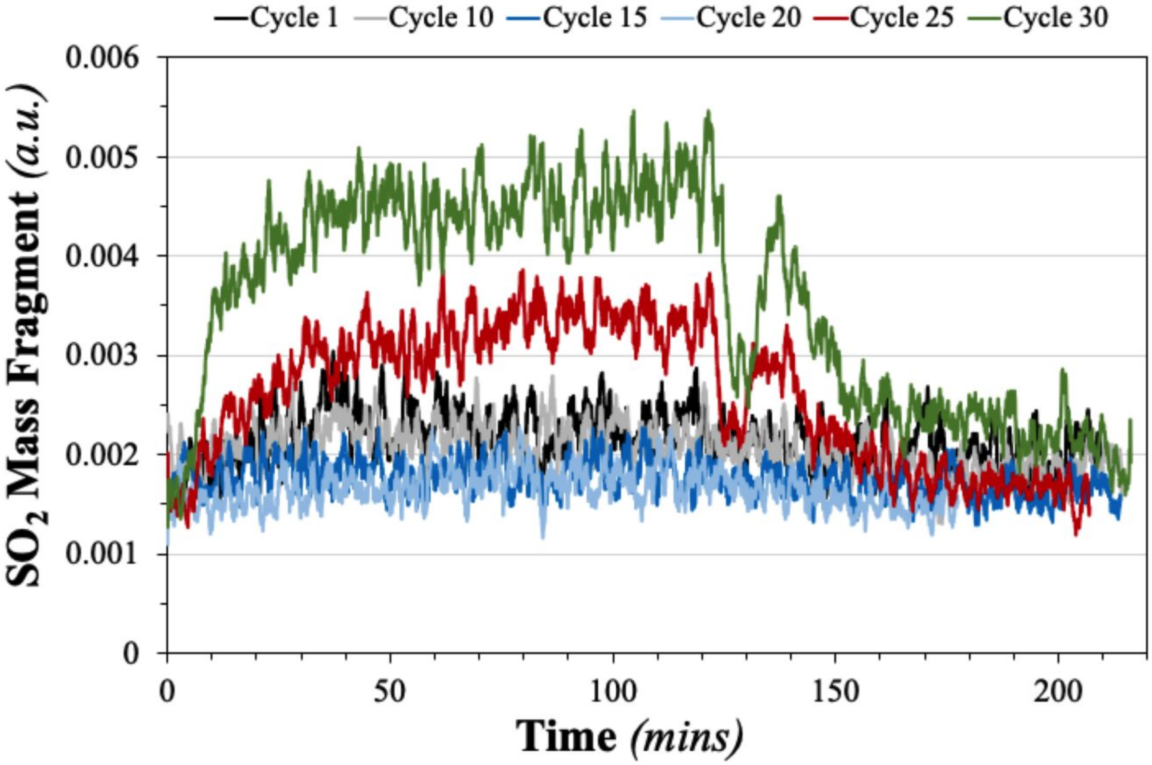

Figure S2. Mass spec breakthrough curves for SO_2 (m/z 64) normalized against Ar (m/z 36) during the absorption/desorption cycles where [P_{66614}][Benzim] and [P_{66614}][Tetz] are exposed to a feed of 14% CO_2 and 0.2% SO_2 in Ar at 22 °C for 2 h, and a desorption period at 90 °C under Ar for 2 h.

[P_{66614}][Benzim]: ^{1}H NMR (500 MHz, DMSO–d_6): δ (ppm) = 0.36 (s, 12H, CH_3(P)), 0.63-0.79 (m, 48H, CH_2(P)), 1.03 (s, 8H, CH_2(P)), 6.30 (s, 2H, C4 and C5), 6.88 (s, 2H, C3 and C6), 7.28 (s, 1H, C1); ^{13}C NMR (126 MHz, DMSO–d_6): δ (ppm) = 13.17, 16.75, 18.59, 20.44, 21.61, 29.01, 30.26, 31.21, 54.92, 115.15, 116.98, 143.95, 148.78. Cl <5 ppm, H_2O <0.1 wt.%.

[P_{66614}][Benzim] (*post exposure*)**:** ^{1}H NMR (500 MHz, DMSO–d_6): δ (ppm) = 0.25-0.33 (s, 12H, CH_3(P)), 0.62-0.72 (m, 48H, CH_2(P)), 1.39 (s, 8H, CH_2(P)), 6.41 (s, 2H, C4 and C5), 7.00 (s, 2H, C3 and C6), 7.48 (s, 1H, C1); ^{13}C NMR (126 MHz, DMSO–d_6): δ (ppm) = 13.06, 16.75, 20.39, 21.35, 28.72, 30.04, 30.97, 114.71, 118.57, 140.48, 144.08.

[P_{66614}][Tetz]: ^{1}H NMR (500 MHz, DMSO–d_6): δ (ppm) = 0.37 (s, 12H, CH_3(P)), 0.78-0.89 (m, 48H, CH_2(P)), 1.96 (s, 8H, CH_2(P)), 7.72 (s, 1H, C1); ^{13}C NMR (126 MHz, DMSO–d_6): δ (ppm) = 13.03, 17.33, 20.58, 21.46, 28.89, 30.10, 31.06, 146.57. Cl <5 ppm, H_2O <0.1 wt.%.

[P_{66614}][Tetz] (*post exposure*)**:** ^{1}H NMR (500 MHz, DMSO–d_6): δ (ppm) = 0.52 (s, 12H, CH_3(P)), 0.92-1.14 (m, 48H, CH_2(P)), 1.99 (s, 8H, CH_2(P)), 7.77-7.97 (s, 1H, C1); ^{13}C NMR (126 MHz, DMSO–d_6): δ (ppm) = 13.48, 17.39, 20.61, 21.75, 28.14, 29.08, 30.34, 31.28, 147.13.

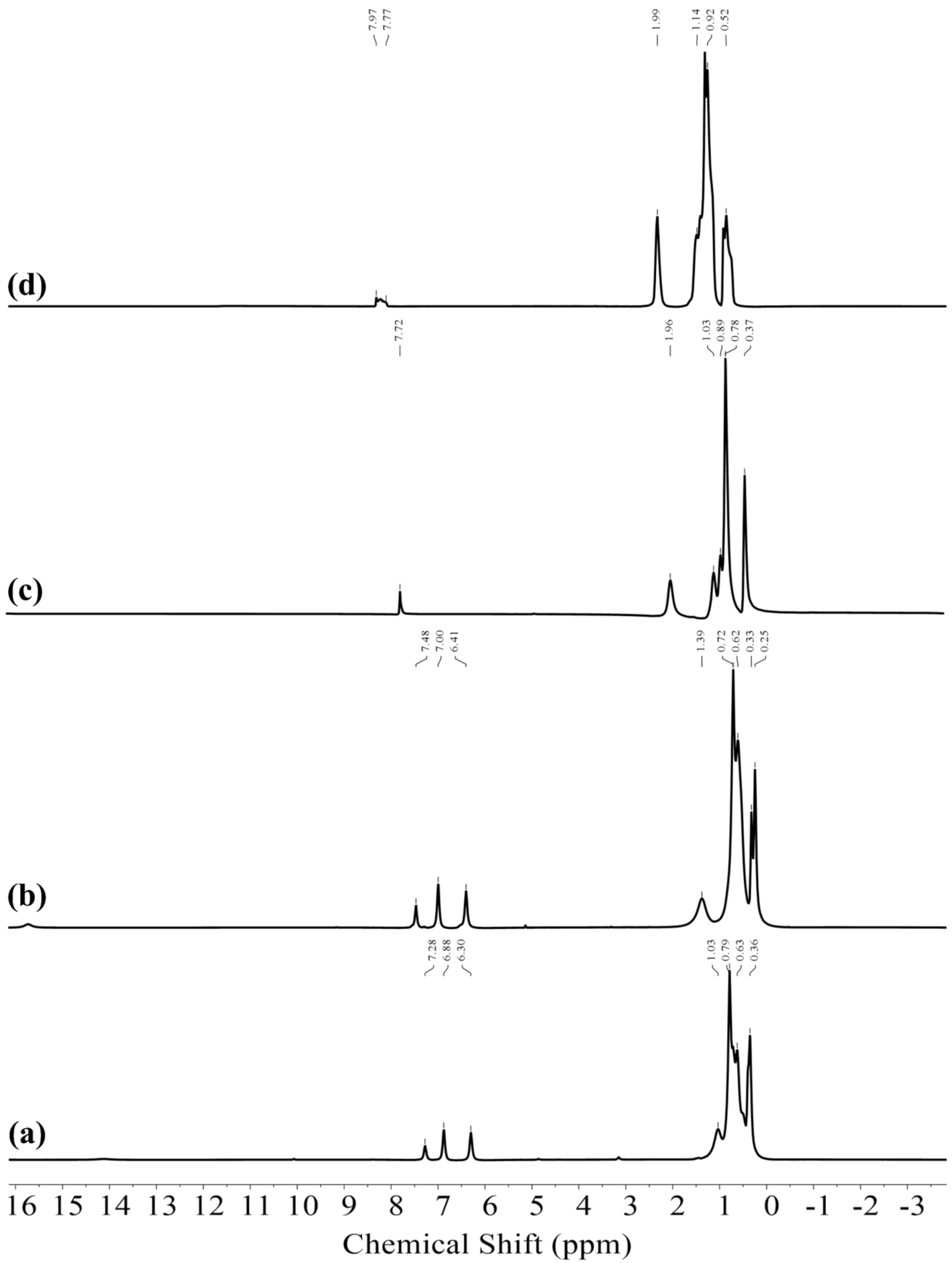

Figure S3. ^{1}H NMR spectra of [P_{66614}][Benzim] before (a) and after (b) exposure, and [P_{66614}][Tetz] before (c) and after (d) exposure (30 cycles of a 2 h absorption at 22 °C under a feed of 14% CO_2 and 0.2% SO_2 in Ar, and a 2 h desorption at 90 °C under Ar).

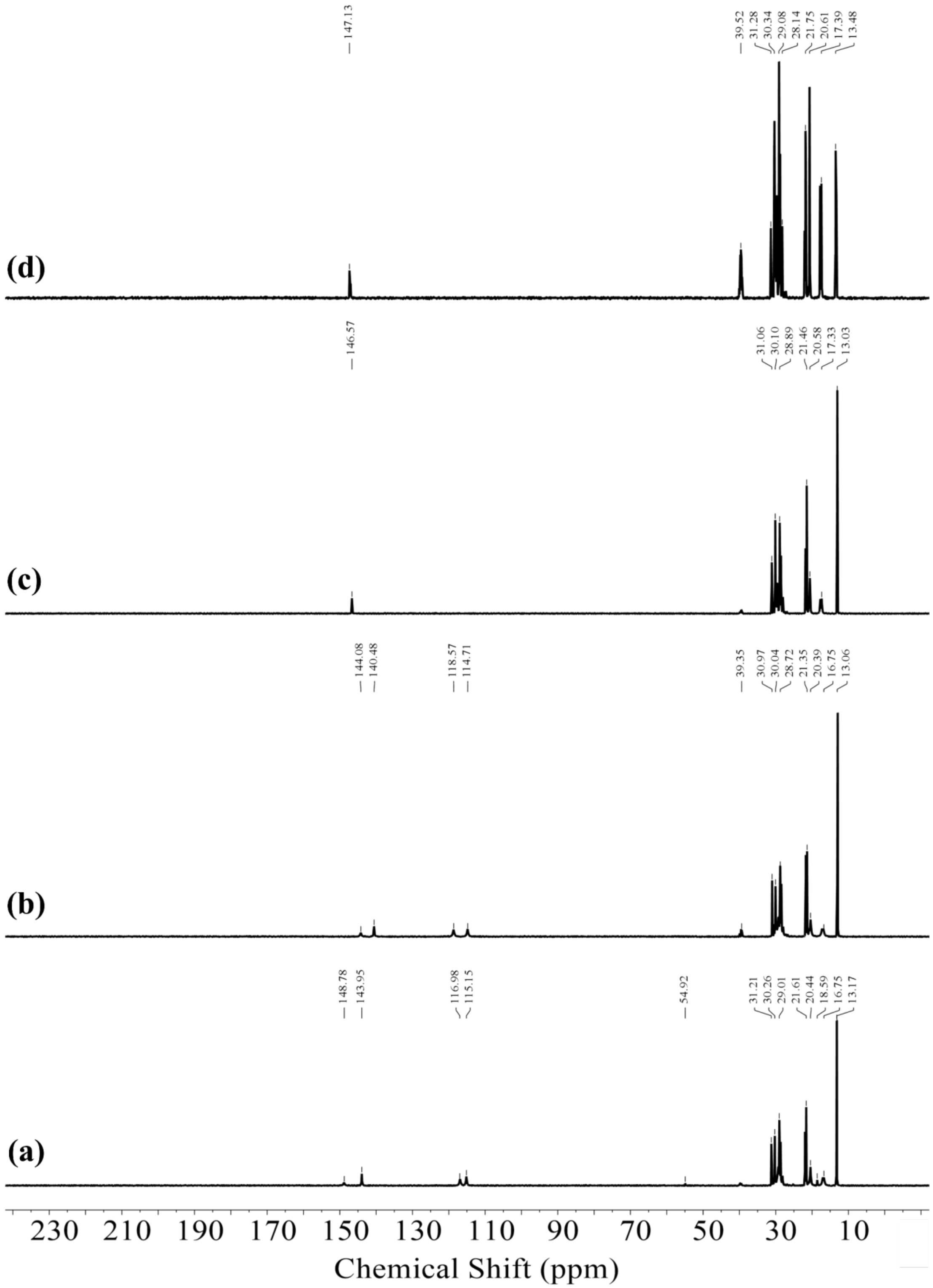

Figure S4. ^{13}C NMR spectra of [P_{66614}][Benzim] before (a) and after (b) exposure, and [P_{66614}][Tetz] before (c) and after (d) exposure (30 cycles of a 2 h absorption at 22 °C under a feed of 14% CO_2 and 0.2% SO_2 in Ar, and a 2 h desorption at 90 °C under Ar).

Table S1. Elemental analysis of ILs before and after exposure in the gas absorption rig.[a]

**Ionic Liquid**		**Elemental Analysis (wt.%)**[a]				
		C	*H*	*N*	*P*	*S*
**[P_{66614}][Benzim]**	*Before*	76.58	12.01	5.38	6.03	nd[b]
	After	72.47	11.73	5.27	8.89	1.64
**[P_{66614}][Tetz]**	*Before*	69.75	12.81	11.09	6.35	nd[b]
	After	69.64	12.79	11.20	6.37	nd[b]

[a]*u*(element) = 0.3 wt % ; [b] nd : not detected.
